# Integrating Tumor Sequencing Into Clinical Practice for Patients With Mismatch Repair-Deficient Lynch Syndrome Spectrum Cancers

**DOI:** 10.14309/ctg.0000000000000397

**Published:** 2021-08-16

**Authors:** Katherine Dixon, Mary-Jill Asrat, Angela C. Bedard, Kristin Binnington, Katie Compton, Carol Cremin, Nili Heidary, Zoe Lohn, Niki Lovick, Mary McCullum, Allison Mindlin, Melanie O'Loughlin, Tammy Petersen, Cheryl Portigal-Todd, Jenna Scott, Genevieve St-Martin, Jennifer Thompson, Ruth Turnbull, Sze Wing Mung, Quan Hong, Marjorie Bezeau, Ian Bosdet, Tracy Tucker, Sean Young, Stephen Yip, Gudrun Aubertin, Katherine A. Blood, Jennifer Nuk, Sophie Sun, Kasmintan A. Schrader

**Affiliations:** 1 Department of Medical Genetics, University of British Columbia, Vancouver, British Columbia, Canada;; 2 Hereditary Cancer Program, BC Cancer, Vancouver, British Columbia, Canada;; 3 Laboratory of Transdisciplinary Research in Genetics, Medicines and Social Sciences, Sherbrooke's University Hospital Center of Clinical Research, Quebec, Canada;; 4 Department of Pathology and Laboratory Medicine, University of British Columbia, Vancouver, British Columbia, Canada;; 5 Department of Medical Genetics, Vancouver Island Health Authority, Victoria, British Columbia, Canada;; 6 Department of Medical Oncology, BC Cancer, Vancouver, British Columbia, Canada.

## Abstract

Uninformative germline genetic testing presents a challenge to clinical management for patients suspected to have Lynch syndrome, a cancer predisposition syndrome caused by germline variants in the mismatch repair (MMR) genes or *EPCAM*.

**METHODS:**

Among a consecutive series of MMR-deficient Lynch syndrome spectrum cancers identified through immunohistochemistry-based tumor screening, we investigated the clinical utility of tumor sequencing for the molecular diagnosis and management of suspected Lynch syndrome families. MLH1-deficient colorectal cancers were prescreened for BRAF V600E before referral for genetic counseling. Microsatellite instability, *MLH1* promoter hypermethylation, and somatic and germline genetic variants in the MMR genes were assessed according to an established clinical protocol.

**RESULTS:**

Eighty-four individuals with primarily colorectal (62%) and endometrial (31%) cancers received tumor-normal sequencing as part of routine clinical genetic assessment. Overall, 27% received a molecular diagnosis of Lynch syndrome. Most of the MLH1-deficient tumors were more likely of sporadic origin, mediated by *MLH1* promoter hypermethylation in 54% and double somatic genetic alterations in *MLH1* (17%). MSH2-deficient, MSH6-deficient, and/or PMS2-deficient tumors could be attributed to pathogenic germline variants in 37% and double somatic events in 28%. Notably, tumor sequencing could explain 49% of cases without causal germline variants, somatic *MLH1* promoter hypermethylation, or somatic variants in *BRAF*.

**DISCUSSION:**

Our findings support the integration of tumor sequencing into current Lynch syndrome screening programs to improve clinical management for individuals whose germline testing is uninformative.

## INTRODUCTION

Lynch syndrome is the most common form of hereditary colorectal cancer (CRC), accounting for 3% of all CRC diagnoses. Lynch syndrome is caused by constitutional, or germline, variants in 1 of 4 genes involved in mismatch repair (MMR), *MLH1*, *MSH2*, *MSH6*, and *PMS2*, or a deletion in *EPCAM* (1,2). Carriers have an estimated 52%–82% risk for CRC and 25%–60% risk for endometrial cancer (EC) by 70 years of age and increased risks for several other cancer types (3). Because of the significant lifetime risk for cancer, identification and molecular diagnosis have critical implications for cancer prevention and early cancer detection through increased endoscopic surveillance, prophylactic intervention, and cascade carrier testing in relatives.

Around 90% of Lynch syndrome-related tumors show deficient MMR (dMMR) characterized by microsatellite instability (MSI) and abnormal MMR protein expression (4,5). This feature is less common in sporadic cancers, observed in 15% of CRC and 3.8% of cancers overall (6–9). Consequently, universal screening of CRC and EC tumors for dMMR has become routine to identify individuals who may benefit from clinical intervention and cancer risk management (10,11). Tumor screening also has therapeutic significance because solid tumors with MMR deficiency are sensitive to immune checkpoint blockades because of their potential for encoding many tumor-specific antigens (12,13).

Although universal screening is both clinically meaningful and cost-effective, only 25%–67% of individuals with dMMR CRC receive a molecular diagnosis of Lynch syndrome (5,14). When no causal germline variants are found, depending on personal and family cancer history, individuals with uninformative germline testing may receive recommendations based on the Lynch syndrome screening guidelines that include early and intensive cancer surveillance. These cases, referred to as Lynch-like syndrome, could represent true Lynch syndrome-related cancers associated with germline MMR gene variants that are cryptic to current technologies, dMMR cancers associated with biallelic somatic variants/aberrations in the MMR or other pathway-related genes, or, more rarely, on the basis of pathogenic germline variants in other cancer predisposition genes (e.g., *POLE*/*POLD1* and *MUTYH*) (15–17). Phenotypic and pathological variability among individuals with Lynch-like syndrome further indicates that this represents a heterogenous clinical entity that may show differential benefit from increased screening (18,19). Double somatic variants in the MMR genes underlie 50%–70% of dMMR tumors that are unexplained by germline variants, somatic *MLH1* hypermethylation, or somatic variants in the proto-oncogene *BRAF* (20–24). Thus, to investigate the clinical utility of tumor sequencing in the molecular diagnosis and clinical management of suspected Lynch syndrome families, we analyzed dMMR Lynch syndrome spectrum tumors by targeted tumor-normal sequencing and described the integration of tumor sequencing into an existing Lynch syndrome assessment protocol of a population-based hereditary cancer program.

## METHODS

### Participants

Individuals were eligible for tumor-normal molecular testing if they received a diagnosis of a MMR-deficient Lynch syndrome spectrum cancer, including colorectal, endometrial, gastric, ovarian, pancreatic, ureter and renal pelvis, biliary tract, brain, sebaceous gland, keratoacanthoma, and small bowel. MMR deficiency, defined as abnormal expression of MLH1, MSH2, MSH6, and/or PMS2, was evaluated by immunohistochemistry (IHC) according to the standard clinical protocol. When indicated, clinical testing for somatic BRAF V600E was performed by IHC or quantitative polymerase chain reaction. Written informed consent for testing was provided from index cases or next of kin between June 2018 and December 2019. This study was approved by the University of British Columbia Clinical Research Ethics Board (H19-02520).

### Tumor and germline testing

Paired tumor-normal testing was performed using the TumorNext-Lynch assay (Ambry Genetics, Aliso Viejo, CA) as previously described (25). Briefly, germline and tumor DNA were extracted from peripheral blood and formalin-fixed paraffin-embedded tumor biopsy specimens, respectively. Capture-based targeted sequencing of *MLH1*, *MSH2*, *MSH6*, *PMS2*, and *EPCAM* was performed, requiring a minimum depth of 20× for germline analysis and 100× for tumor analysis. MSI, small variants, and copy number alterations were called according to a published bioinformatic pipeline (25). For *EPCAM*, only gross deletions encompassing the 3' end were reported. Germline variants were confirmed by Sanger sequencing, and constitutional methylation was assessed through orthogonal clinical testing when indicated.

### Statistical analysis and interpretation

Lynch syndrome cases were identified by findings of pathogenic or likely pathogenic germline variants in *MLH1*, *MSH2*, *MSH6*, *PMS2*, or *EPCAM*, including constitutional methylation of the *MLH1* promoter. Likely sporadic cancers were defined as cases without causal germline variants and presumed biallelic somatic events affecting genes that were at least partially concordant with findings from IHC, including *MLH1* promoter hypermethylation or double somatic genetic alterations. These included cases with 2 somatic pathogenic or likely pathogenic variants or cases with 1 somatic pathogenic or likely pathogenic variant and somatic copy loss or copy neutral loss of heterozygosity (LOH). Statistical analysis was performed in R. Descriptive statistics were summarized by percentages for categorical variables and by median and range for continuous variables.

## RESULTS

Targeted tumor sequencing was performed for 84 individuals referred for clinical genetic assessment on the basis of a dMMR Lynch syndrome spectrum cancer examined by IHC (Table 1). According to the standard clinical protocol, BRAF V600E IHC or quantitative polymerase chain reaction was performed in MLH1-deficient colorectal tumors before or at the time of referral to exclude likely sporadic cancers. Most of the tumors were of primary colorectal (62%, n = 52) or endometrial (31%, n = 26) origin but also included sebaceous adenomas (n = 2), small bowel cancer (n = 2), gastric cancer (n = 1), and renal cell carcinoma (n = 1). Seventy percent (n = 59) of individuals met the revised Bethesda criteria for clinical genetic testing, and an additional 7 (8%) met the Amsterdam I or II criteria on the basis of multiple primary tumors and/or family history of multiple Lynch syndrome spectrum cancers.

**Table 1.** Study population demographics

	Index cases, N (%)
Total	84
Cancer type	
Colorectal	52 (62)
Endometrial	26 (31)
Other	6 (7.1)
Biological sex	
Female	50 (60)
Male	34 (40)
Age at diagnosis	
≤50	21 (25)
>50	63 (75)
TNM stage	
I	21 (25)
II	7 (8.3)
III	33 (39)
IV	9 (11)
Unknown	14 (17)
Clinical criteria	
Amsterdam	7 (8.3)
Bethesda	59 (70)
None	18 (21)
IHC status	
MLH1/PMS2	39 (46)
MSH2/MSH6	22 (26)
MSH6	9 (11)
PMS2	10 (12)
MLH1/PMS2/MSH6	2 (2.4)
MSH6/PMS2	2 (2.4)

IHC, immunohistochemistry; TMN, tumor, node, metastasis.

**Figure 1.**
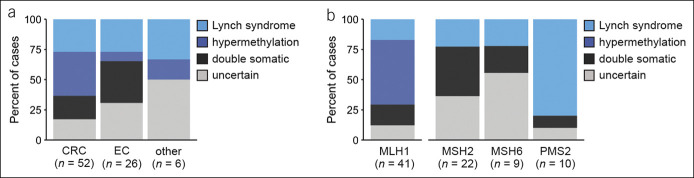
Predicted origin of dMMR tumors analyzed by tumor sequencing. (**a**) Percent of CRC, EC, and other cancer types resulting from pathogenic or likely pathogenic germline variants, *MLH1* promoter hypermethylation, double somatic events, or that remain unexplained. (**b**) Predicted molecular origin of dMMR tumors by immunohistochemistry status. MLH1: combined MLH1/PMS2 loss; MSH2: combined MSH2/MSH6 loss; MSH6: MSH6 loss with normal MSH2 expression; and PMS2: PMS2 loss with normal MLH1 expression. Two tumors associated with MSH6/PMS2 deficiency, 1 germline, and 1 unexplained are not shown. CRC, colorectal cancer; dMMR, deficient mismatch repair; EC, endometrial cancer.

MSI, *MLH1* promoter hypermethylation, and germline and somatic variants were analyzed concurrently using Ambry Genetics' TumorNext-Lynch assay (Figure 1, see Supplementary Figure 1 and Supplementary Table 1, Supplementary Digital Content 1, http://links.lww.com/CTG/A667) (25). This included single-nucleotide variants, small insertions and deletions, copy number alterations, and LOH; orthogonal clinical testing for constitutional methylation of the *MLH1* promoter was also performed when indicated by the phenotype of the index case and IHC status. Overall, 27% (n = 23) of individuals received a molecular diagnosis of Lynch syndrome. Pathogenic and likely pathogenic germline variants were identified in 27% (14/52) and 27% (7/26) of individuals with CRC and EC, respectively, and in 2 individuals with other dMMR cancers within the Lynch syndrome spectrum. A molecular diagnosis was determined for 5 of 7 cases meeting the Amsterdam I or II criteria; however, 2 carriers (9%) did not meet any Amsterdam I, Amsterdam II, or revised Bethesda testing criteria.

Tumors with abnormal expression of MLH1 by IHC were most likely to be of somatic origin because 54% (22/41) were attributed to *MLH1* promoter hypermethylation and 17% (7/41) were attributed to double somatic events in *MLH1*. Notably, methylation of the *MLH1* promoter was found in 3 of 7 MLH1-deficient Lynch syndrome–related tumors. Tumor-specific methylation was found in a CRC and small bowel cancer for 2 *PMS2* carriers, and constitutional methylation of *MLH1* was found in 1 individual with EC who met the Amsterdam I criteria (see Supplementary Figures 2 and 3, Supplementary Digital Content 1, http://links.lww.com/CTG/A667). Double somatic alterations ultimately explained 58% (7/12) of MLH1-deficient cases without promoter hypermethylation or causal germline variants.

Among tumors with abnormal IHC for MSH2, MSH6, and/or PMS2, pathogenic germline variants were identified in 37% (16/43). Tumor sequencing identified double somatic events in an additional 28% (n = 12), indicating a likely sporadic cancer occurrence in 44% (12/27) of cases without causal germline variants. More than one-third (35%; n = 15) of MSH2-deficient, MSH6-deficient, and/or PMS2-deficient tumors could not be explained by somatic or germline genetic alterations. Greater uncertainty was revealed for 1 individual with MSH2/MSH6-deficient EC associated with a single somatic variant in *MSH2* identified by tumor-normal sequencing and a personal history of MMR-proficient CRC. Repeat IHC of the colorectal and endometrial tumors did not suggest loss of MSH6 expression, as originally seen in the context of abnormal MSH2 IHC of the patient's EC. These results were interpreted as possible tumor heterogeneity or as a possible false positive finding of MMR deficiency. Methylation of *MSH2*, *MSH6*, and *PMS2* was not assessed in this study.

**Figure 2.**
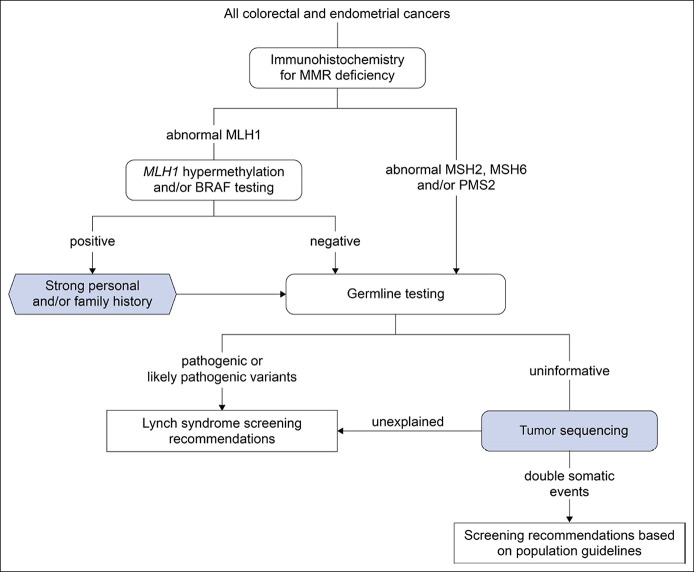
Modified framework for universal Lynch syndrome screening.

Across this series of dMMR Lynch syndrome spectrum cancers, double somatic genetic alterations explained 23% (n = 19) of cases overall and 49% (19/39) of those lacking causal germline variants, *MLH1* promoter hypermethylation, and BRAF V600E, also known as Lynch-like syndrome. Likely sporadic cancers caused by *MLH1* promoter hypermethylation were associated with an older age at first cancer diagnosis compared with individuals with Lynch syndrome (median 71.5 vs 52 years, *P* = 5.3 × 10^−4^, Wilcoxon rank sum test) and a lower proportion of patients had Prediction Model for Gene Mutations (PREMM_5_) scores ≥2.5% (32% vs 78%, *P* = 2.7 × 10^−3^, Fisher exact test). No differences in clinicopathologic characteristics were observed between cancers associated with double somatic MMR variants and cases that remained molecularly unexplained. Therefore, consistent with the recent European guidelines, our findings support integrating tumor sequencing secondary to germline testing in the diagnostic odyssey of Lynch and Lynch-like syndrome (Figure 2) (26). Given uninformative results from germline testing, secondary tumor sequencing would be indicated for 46% (n = 36, 95% confidence interval 35%–58%) of referrals to our clinic for dMMR CRC or EC identified through IHC-based tumor screening (Table 2). Germline testing would not be excluded on the basis of *MLH1* hypermethylation or BRAF V600E for individuals with a personal and/or family history suggestive of Lynch syndrome. However, 1 *PMS2* carrier with late-onset MLH1-deficient CRC and without a family history of CRC would have been missed, given the finding of *MLH1* promoter hypermethylation.

**Table 2.** Comparison of secondary tumor sequencing with germline-only testing for the clinical management of MMR-deficient colorectal and ECs identified by universal IHC-based screening

	Molecular diagnosis of LS, n (%)	Likely sporadic cancers, n (%)		Cases that remain unexplained, n (%)		Testing metrics	
		Without TS^a^	With TS^b^	Without TS	With TS	Referrals requiring TS, % (95% CI)^c^	LS cases missed, n (%)
Total (n = 78)	20 (26%)	22 (28%)	41 (53%)	36 (46%)	17 (22%)	46% (35–58%)	1 (5%)
Cancer type							
CRC (n = 52)	13 (25%)	20 (38%)	30 (58%)	19 (37%)	9 (17%)	37% (24–52%)	1 (7%)
EC (n = 26)	7 (27%)	2 (8%)	11 (42%)	17 (65%)	8 (31%)	65% (44–83%)	0
Clinical testing criteria							
Amsterdam I/II (n = 7)	5 (71%)	1 (14%)	1 (14%)	1 (14%)	1 (14%)	14% (0–58%)	0
Revised Bethesda (n = 54)	13 (24%)	16 (30%)	29 (54%)	25 (46%)	12 (22%)	46% (33–60%)	1 (7%)
None (n = 17)	2 (12%)	5 (29%)	11 (65%)	10 (59%)	4 (29%)	59% (33–82%)	0
PREMM_5_ score							
<2.5% (n = 33)	3 (9%)	15 (45%)	23 (70%)	15 (45%)	7 (21%)	45% (28–64%)	1 (25%)
≥2.5% (n = 45)	17 (38%)	7 (16%)	18 (40%)	21 (47%)	10 (22%)	47% (32–62%)	0

CI, confidence interval; CRC, colorectal cancer; EC, endometrial cancer; IHC, immunohistochemistry; LS, Lynch syndrome; MMR, mismatch repair; PREMM, Prediction Model for Gene Mutations; TS, tumor sequencing.

a The current testing algorithm without tumor sequencing includes sequential IHC for MMR proteins, BRAF V600E IHC in MLH1-deficient CRCs, *MLH1* promoter methylation testing in MLH1-deficient ECs and *BRAF* wild-type CRCs, and germline testing in tumors without *MLH1* promoter hypermethylation or BRAF V600E.

b The modified testing algorithm, described in Figure 2, includes targeted tumor sequencing of *MLH1*, *MSH2*, *MSH6*, *PMS2*, and *EPCAM* secondary to germline testing when the results are uninformative.

c Referrals requiring tumor sequencing are defined as the proportion of referrals for genetic counseling and germline testing, based on previous abnormal IHC, lack of *MLH1* promoter hypermethylation, and normal BRAF IHC, anticipated to have uninformative results from germline genetic testing.

After targeted tumor-normal sequencing, 17% (n = 9) and 31% (n = 8) of dMMR CRC and EC, respectively, remained unexplained. Single somatic variants that could partially explain the results from IHC-based screening were identified in 65% (11/17) while double somatic variants discordant with IHC were identified in 1 case. Discordance between IHC and mutation status has been recognized by others in the context of isolated PMS2 loss in individuals without causal germline variants in *PMS2* (27,28). In this series, biallelic somatic alterations in *MLH1* (a somatic missense variant and LOH) were found in a CRC with isolated PMS2 deficiency and neither germline nor somatic variants in *PMS2*. These findings suggest an alternate mechanism of PMS2 protein degradation mediated by MLH1 dysfunction rather than MLH1 loss.

## DISCUSSION

Distinguishing sporadic and hereditary cancers has important implications for clinical management of suspected hereditary cancer families. Uninformative germline testing for individuals with pathological, molecular, or phenotypic indications of Lynch syndrome leads to an uncertainty in cancer risk, nonspecific recommendations for cancer screening, and missed opportunities for the use of targeted therapies and cascade carrier testing. Our findings indicate that within a clinical setting, double somatic genetic alterations account for almost half of dMMR cancers that remain unexplained by pathogenic or likely pathogenic germline variants, *MLH1* promoter hypermethylation, or BRAF V600E. Integrating tumor sequencing into current clinical testing algorithms may thus avoid unnecessary harm for individuals with low-to-moderate cancer risk.

Tumor sequencing has reformed precision cancer medicine by identifying genetic markers with diagnostic, prognostic, and therapeutic significance among molecularly heterogenous tumors (29,30). When followed by site-specific germline testing, panel-based tumor sequencing also allows the ascertainment of previously unknown hereditary cancer families. Notably, compared with IHC-based and MSI-based Lynch syndrome screening, upfront tumor sequencing has shown comparable sensitivity, specificity, and clinical validity for identifying dMMR tumors and for the molecular diagnosis of Lynch syndrome (31). This approach could overcome the complexity of current testing algorithms that often involve iterative testing and multiple care providers. The current costs of sequencing may prevent universal integration in public healthcare systems due to the limited funding, availability of laboratory, clinical and genetic counseling personnel, and lack of consensus guidelines among regional health authorities (32,33). However, incorporating tumor sequencing secondary to germline genetic testing for Lynch syndrome would allow its efficient integration into existing testing algorithms.

Despite recent advances in sequencing technologies and widespread implementation of clinical multigene panels, many individuals receive uninformative results from germline genetic testing. In this study, we could not determine causation for 22% of dMMR CRC and EC cases, including the 1 individual meeting Amsterdam I criteria. This may reflect in part the limitations of targeted next-generation sequencing for detecting certain types of germline variations, such as copy number neutral structural variants, complex rearrangements, or regulatory variants. For example, causal inversions in *MSH2* that eluded conventional diagnostic assays have been found in some Lynch syndrome families (34,35). Variants of uncertain significance also remain challenging in the clinical genetic setting, with reported rates of almost 40% in individuals undergoing panel-based multigene sequencing (36,37). Through the detection of single somatic variants or LOH, the potential for tumor sequencing to inform variant interpretation was demonstrated for 1 variant identified in this cohort, *MSH2* c.1829A > C (p.H610P) (38). Although initially classified as a variant of uncertain significance at the time of referral, this variant was subsequently reclassified as likely pathogenic based on its association with MSH2 protein deficiency, MSI, and tumor LOH in multiple individuals with strong personal and family history of Lynch syndrome spectrum cancers. Further discussion is needed regarding the meaningful integration of molecular tumor data for exploring potential disease-causing variation in individuals with phenotypic indications of high-penetrance cancer syndromes.

Although the prevalence of double somatic variants reported here is consistent with previous studies, this study was small in size and was limited to cancers screened at a single center (21–24). Universal CRC and EC screening for MMR deficiency has not yet been broadly adopted across British Columbia, and screening protocols for MMR deficiency, somatic *BRAF* variants, and *MLH1* promoter methylation vary between regional health authorities. Therefore, our findings may not be representative of true population-based tumor screening programs. Despite prescreening for BRAF V600E, *MLH1* promoter hypermethylation was found in most of the MLH1-deficient CRC cases, supporting a hybrid approach for excluding likely sporadic cancers through sequential methylation analysis and BRAF testing (39). Rare tumors with global hypermutation have also been explained by germline and somatic variants in genes encoding subunits of DNA polymerase, including *POLD1* and *POLE* (16,40). Incorporating other known driver genes or mutational hotspots into targeted tumor sequencing panels could also allow the evaluation of alternative causes of tumorigenesis.

Within our cohort, biallelic somatic genetic variants in the MMR genes indicated a likely sporadic cancer occurrence in around half of Lynch-like syndrome cases identified by IHC-based tumor screening. Consistent with evolving clinical practice, these findings helped exclude the possibility of Lynch Syndrome and allowed management recommendations to become more confidently tailored toward the patient's personal and family history (41). In BC, residual screening recommendations are made in accordance with provincial CRC screening guidelines for average-risk and moderate-risk individuals (42). Patients whose cancer remained unexplained after tumor-normal sequencing were counseled about the uncertainty of their genetic testing results and given screening recommendations based on personal and family cancer history, which included broader testing when there was clinical indication of alternative high-penetrance cancer susceptibility. Lynch syndrome screening guidelines were recommended for individuals meeting phenotype-based testing criteria for Lynch syndrome while average-risk and moderate-risk provincial CRC screening guidelines were discussed as possible management options for individuals without a personal or family history suggestive of Lynch syndrome or other cancer predisposition syndromes (43). Because the effectiveness of regular EC screening in asymptomatic individuals is uncertain, it was not recommended. Supported by the identification of pathogenic germline variants in 1 sebaceous adenoma and 1 small bowel cancer in this study, screening for MMR deficiency may also be warranted across a broader spectrum of cancer types (44). Future studies are needed to evaluate the feasibility and cost-effectiveness of various testing strategies and the implementation of multidisciplinary programs that integrate pathology, oncology, and clinical genetics.

## CONFLICTS OF INTEREST

**Guarantor of the article:** Kasmintan A. Schrader, MBBS, PhD

**Specific author contributions:** Jennifer Nuk, MSc, Sophie Sun, MD, and Kasmintan A. Schrader, MBBS, PhD, are co-senior authors. J.N., S.S., and K.A.S.: contributed to the conception and design of the study. K.D., M.-J.A., A.C.B., K.B., K.C., C.C., N.H., Z.L., N.L., M.M., A.M., M.O., T.P., C.P.-T., J.S., G.S.-M., J.T., R.T., M.B., I.B., T.T., S. Young, S. Yip, G.A., K.A.B., J.N., S.S., and K.A.S.: contributed to the acquisition of data. K.D., S.W.M., and Q.H.: contributed to the analysis of data. K.D. and K.A.S.: contributed to the interpretation of data and drafted the article. All of the authors revised the article critically for important intellectual content.

**Financial support:** K.A.S. is supported by the Canadian Institutes of Health Research and the Michael Smith Foundation for Health Research.

**Potential competing interests:** None to report.

**Study Highlights**

**WHAT IS KNOWN**

✓ Universal screening for mismatch repair (MMR) deficiency in colorectal and endometrial cancers is an effective strategy for identifying families with Lynch syndrome.
✓ Many individuals receive uninformative results from germline genetic testing.
✓ Somatic variants in the MMR genes have been identified in some MMR-deficient tumors that cannot otherwise be explained.

**WHAT IS NEW HERE**

✓ Tumor-normal sequencing was performed for a consecutive series of patients with MMR-deficient Lynch syndrome spectrum cancers referred to a provincial hereditary cancer clinic.
✓ Causal somatic variants were identified in half of the cases that could not be explained by germline variants, MLH1 promoter hypermethylation, or BRAF V600E.

## Supplementary Material

SUPPLEMENTARY MATERIAL
