# Supplementary material for: Integrating Tumor Sequencing Into Clinical Practice for Patients With Mismatch Repair-Deficient Lynch Syndrome Spectrum Cancers
Source: Clin Transl Gastroenterol. 2021 Aug 16;12(8):e00397. doi: 10.14309/ctg.0000000000000397 (PMC8373535; doi:10.14309/ctg.0000000000000397)
Supplement: SUPPLEMENTARY MATERIAL [file ct9-12-e00397-s001.docx]

**Supplementary Materials**

**Integrating Tumour Sequencing into Clinical Practice for Patients with Mismatch Repair-Deficient Lynch Syndrome Spectrum Cancers**

Katherine Dixon^1^, Mary-Jill Asrat^2^, Angela C. Bedard^2^, Kristin Binnington^2^, Katie Compton^2^, Carol Cremin^2^, Nili Heidary^2^, Zoe Lohn^2^, Niki Lovick^2^, Mary McCullum^2^, Allison Mindlin^2^, Melanie O'Loughlin^2^, Tammy Petersen^2^, Cheryl Portigal-Todd^2^, Jenna Scott^2^, Genevieve St-Martin^2^, Jennifer Thompson^2^, Ruth Turnbull^2^, Sze Wing Mung^2^, Quan Hong^2^, Marjorie Bezeau^2,3^, Ian Bosdet^4^, Tracy Tucker^4^, Sean Young^4^, Stephen Yip^4^, Gudrun Aubertin^5^, Katherine A. Blood^1,2,5^, Jennifer Nuk^1,2^*, Sophie Sun^2,6^*, Kasmintan A. Schrader^1,2^*

^1^ Department of Medical Genetics, University of British Columbia, Vancouver, BC, Canada

^2^ Hereditary Cancer Program, BC Cancer, Vancouver, BC, Canada

^3^ Laboratory of Transdisciplinary Research in Genetics, Medicines and Social Sciences, Sherbrooke's University Hospital Center of Clinical Research, QC, Canada

^4^ Department of Pathology and Laboratory Medicine, University of British Columbia, Vancouver, BC, Canada

^5^ Department of Medical Genetics, Vancouver Island Health Authority, Victoria, BC, Canada

^6^ Department of Medical Oncology, BC Cancer, Vancouver, BC, Canada

*Co-senior authors

**Supplementary Table 1. Demographic and clinical information by predicted cancer origin**

|  | Lynch syndrome | MLH1 promoter hypermethylation | Double somatic variants | Unexplained |
| --- | --- | --- | --- | --- |
| **Total (*n* = 84)** | 23 (27) | 22 (26) | 19 (23) | 20 (24) |
| **Age at earliest diagnosis**, median (range) | 52 (32-86) | 71.5 (33-87) | 53 (28-77) | 55.5 (24-84) |
| **PREMM_5_ score**, median (range) | 7.7 (0.9-50) | 2.2 (0.9-14) | 2.9 (1.7-9.0) | 2.9 (1.2-50) |
| **Clinical testing criteria** |  |  |  |  |
| Amsterdam I/II (*n* = 7) | 5 | 1 | 0 | 1 |
| Revised Bethesda (*n* = 59) | 16 | 15 | 13 | 15 |
| None (*n* = 18) | 2 | 6 | 6 | 4 |
| **PREMM_5_ score** |  |  |  |  |
| < 2.5% (*n* = 35) | 5 | 15 | 8 | 7 |
| ≥ 2.5% (*n* = 49) | 18 | 7 | 11 | 13 |
| **Colorectal (*n* = 52)** | 14 (27) | 19 (37) | 10 (19) | 9 (17) |
| *Sex* |  |  |  |  |
| Female | 5 | 7 | 3 | 6 |
| Male | 9 | 12 | 7 | 3 |
| *TNM stage* |  |  |  |  |
| I/II | 0 | 1 | 2 | 4 |
| III/IV | 12 | 17 | 4 | 4 |
| Unknown | 2 | 1 | 4 | 1 |
| *Histologic grade* |  |  |  |  |
| Well- to moderately-differentiated | 11 | 7 | 5 | 6 |
| Poorly- to undifferentiated | 2 | 12 | 5 | 2 |
| Unknown | 1 | 0 | 0 | 1 |
| **Endometrial (*n* = 26)** | 7 (27) | 2 (8) | 9 (35) | 8 (31) |
| *TNM stage* |  |  |  |  |
| I/II | 4 | 2 | 6 | 6 |
| III/IV | 1 | 0 | 2 | 1 |
| Unknown | 2 | 0 | 1 | 1 |
| *FIGO grade* |  |  |  |  |
| 1 | 6 | 1 | 4 | 4 |
| 2-3 | 1 | 1 | 5 | 3 |
| Unknown | 0 | 0 | 0 | 1 |
| **Other (*n* = 6)** | 2 (33) | 1 (17) | 0 | 3 (50) |

**
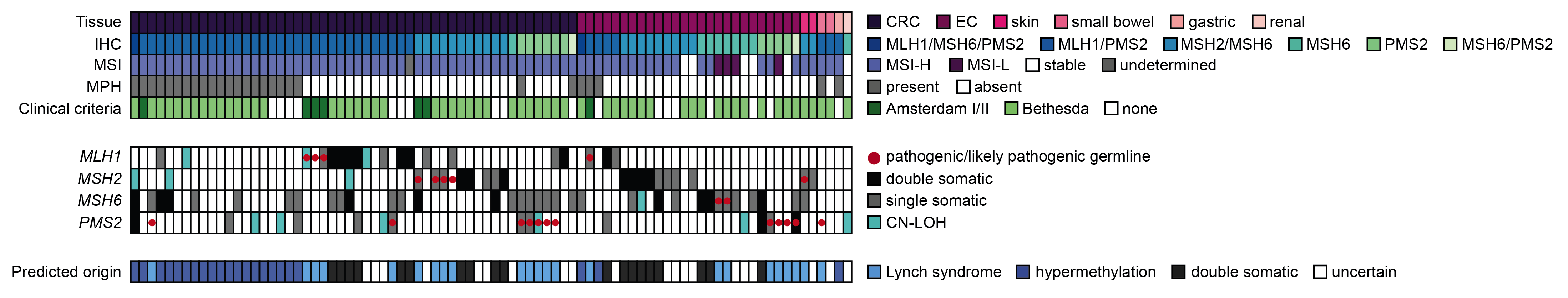
**

**Supplementary Figure 1. Tumour characteristics and sequencing results for dMMR Lynch syndrome spectrum cancers identified by IHC-based tumour screening.** CN-LOH, copy neutral loss of heterozygosity; CRC, colorectal cancer; EC, endometrial cancer; IHC, immunohistochemistry; MPH, *MLH1* promoter hypermethylation; MSI, microsatellite instability; MSI-H, MSI high; MSI-L, MSI low.


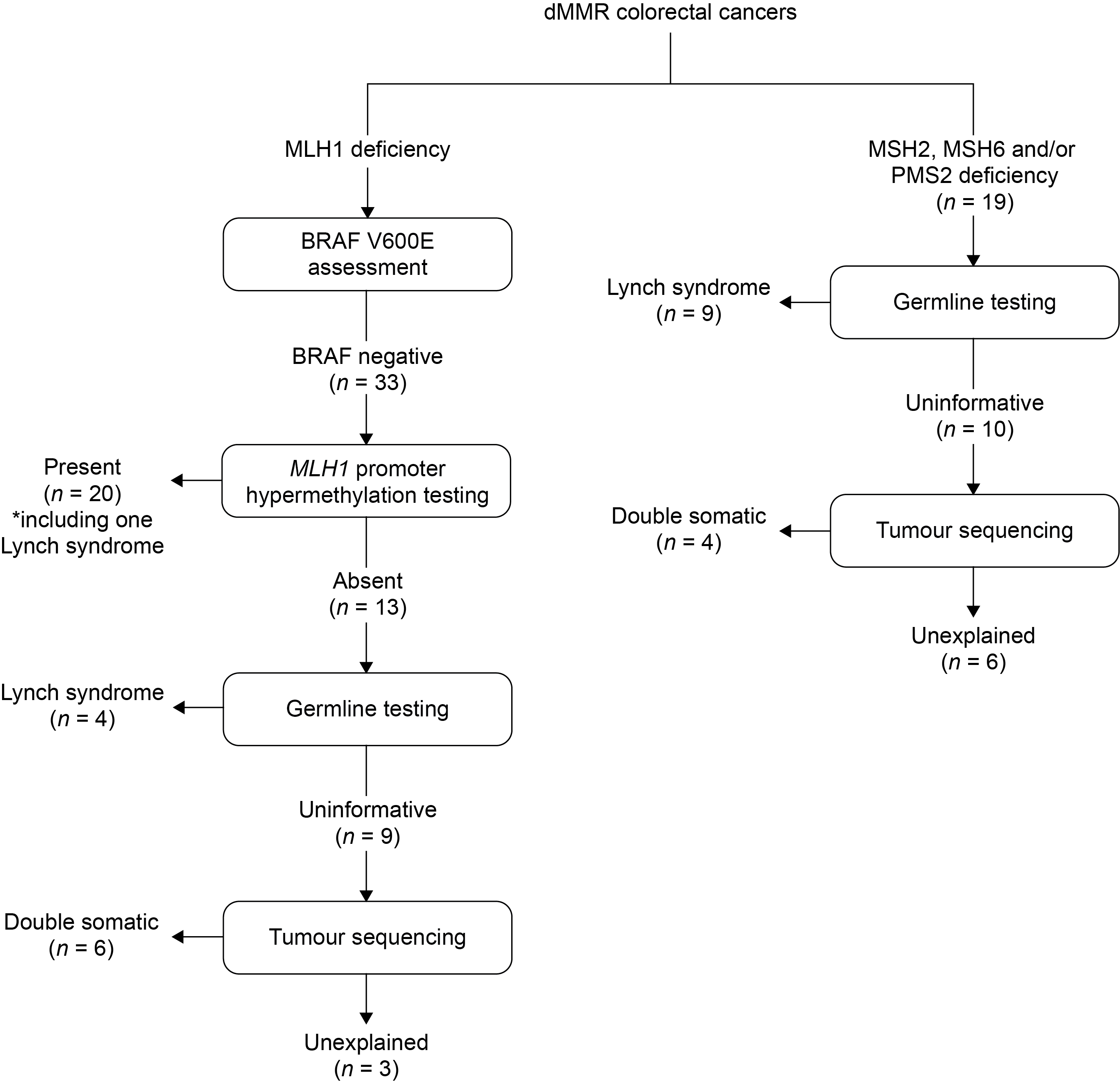


**Supplementary Figure 2. Updated testing algorithm for dMMR colorectal cancers and distribution of cases based on data from the current study.** *One *PMS2* carrier would not have been referred for germline genetic testing based on *MLH1* promoter methylation in their tumour and absence of a personal or family history suggestive of Lynch syndrome.


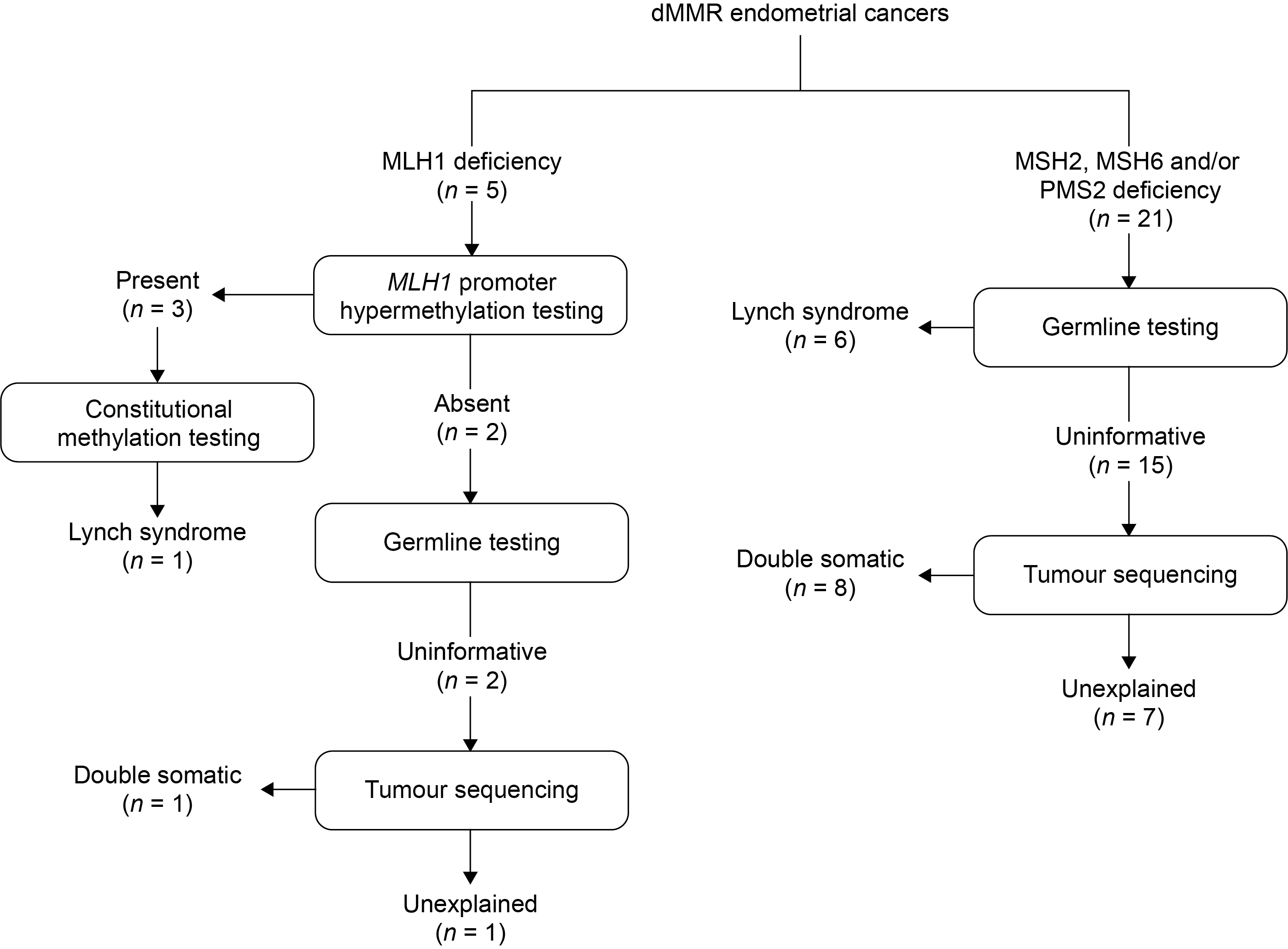


**Supplementary Figure 3. Updated testing algorithm for dMMR endometrial cancers and distribution of cases based on data from the current study.** *Testing for constitutional methylation was indicated for one individual meeting Amsterdam I criteria.
